# Supplementary material for: Controlled alignment of supermoiré lattice in double-aligned graphene heterostructures
Source: Nat Commun. 2023 Jul 12;14:4142. doi: 10.1038/s41467-023-39893-5 (PMC10338430; doi:10.1038/s41467-023-39893-5)
Supplement: Supplementary file 1 — Supplementary Information [file 41467_2023_39893_MOESM1_ESM.pdf]

SUPPLEMENTARY INFORMATION FOR

Controlled alignment of supermoiré lattice in double-aligned graphene heterostructures

Junxiong Hu[1,2#], Junyou Tan[2#], Mohammed M. Al Ezzi[1,2#], Udvas Chattopadhyay[1,2], Jian Gou[1], Yuntian Zheng[1], Zihao Wang[3,4], Jiayu Chen[1], Reshmi Thottathil[1], Jiangbo Luo[1], Kenji Watanabe[5], Takashi Taniguchi[6], Andrew Thye Shen Wee[1], Shaffique Adam[1,2], Ariando Ariando[1]*

[1]Department of Physics, National University of Singapore, Singapore 117542

[2]Centre for Advanced 2D Materials and Graphene Research Centre, National University of Singapore, Singapore 117551

[3]Department of Materials Science and Engineering, National University of Singapore, Singapore 117575, Singapore

[4]Institute for Functional Intelligent Materials, National University of Singapore, Singapore, Singapore

[5]Research Center for Functional Materials, National Institute for Materials Science, Tsukuba, Japan

[6]International Center for Materials Nanoarchitectonics, National Institute for Materials Science, Tsukuba, Japan.

[#]These authors contributed equally to this work.

[*]Email: ariando@nus.edu.sg

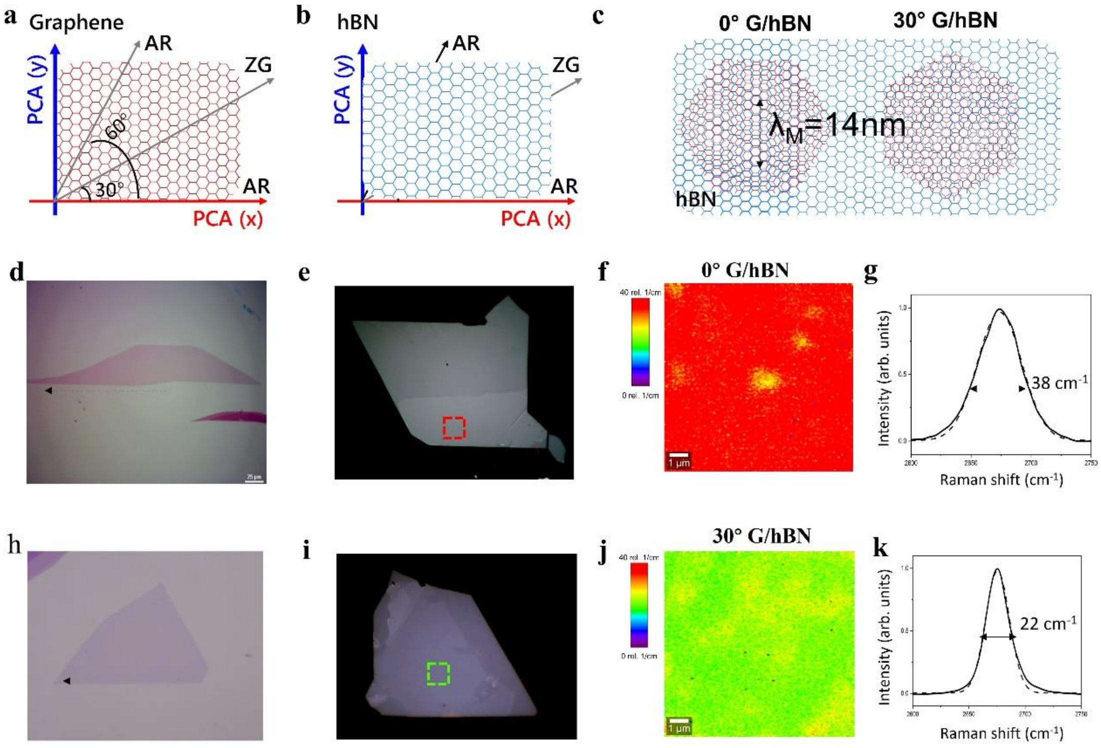

Supplementary Figure 1. Optical alignment of graphene and hBN using a traditional method. Schemic of **a**, graphene, **b**, hBN and **c**, two possible configurations when the alignment of graphene and hBN. **d**, Optical image of graphene with straight edge for alignment. **e**. Optical image of G/hBN stack using the graphene in **d**. **f**, FWHM mapping of Raman 2D-band for the red area in **e**. **g**, Typical Rmana spectra of the 2D band, showing the Gaussian fitting of FWHM of 38 cm^{-1}. **h**, Optical image of graphene with straight edge. **i**, Optical image of G/hBN stack using the graphene in **h. j**, FWHM mapping of Raman 2D-band for the green area in **i**. **k**, Typical Raman spectra of the 2D band, showing the Gaussian fitting of FWHM of 22 cm^{-1}. Graphene and hBN share a similar honeycomb lattice structure, and when hBN is stacking on graphene, the slight lattice mismatch (1.8%) between two crystals produces a hexagonal moiré pattern and a maximum moiré period of ~14 nm can be achieved when the crystallographic lattices are perfectly aligned. According to the crystallographic structures, both graphene and hBN crystals have two possible chirality: Armchair (AR) or zigzag (ZG). When the flake angle is 30°, 90°, and 150°, the two adjacent edges have different chirality (AR/ZG); When the angle is 60°and 120°, both edges have the same chirality (AR/AR or ZG/ZG) (Fig. S1a, b). The uncertain edges chirality of graphene and hBN leads to two possible combinations when stacking together. As illustrated in Fig. S1c, when we align the ZG edge of T-hBN with graphene, there are two possible configurations: ZG-ZG and ZG-AR. The former forms the 0° G/hBN moiré superlattice (Fig. S1d-g) and the latter is equal to rotating the edge of graphene with 30°, named as 30° G/hBN (Fig. S1 h-k). Therefore, when we align the straight edges of graphene and hBN, there are only 50% to achieve the 0° G/hBN moiré superlattice.

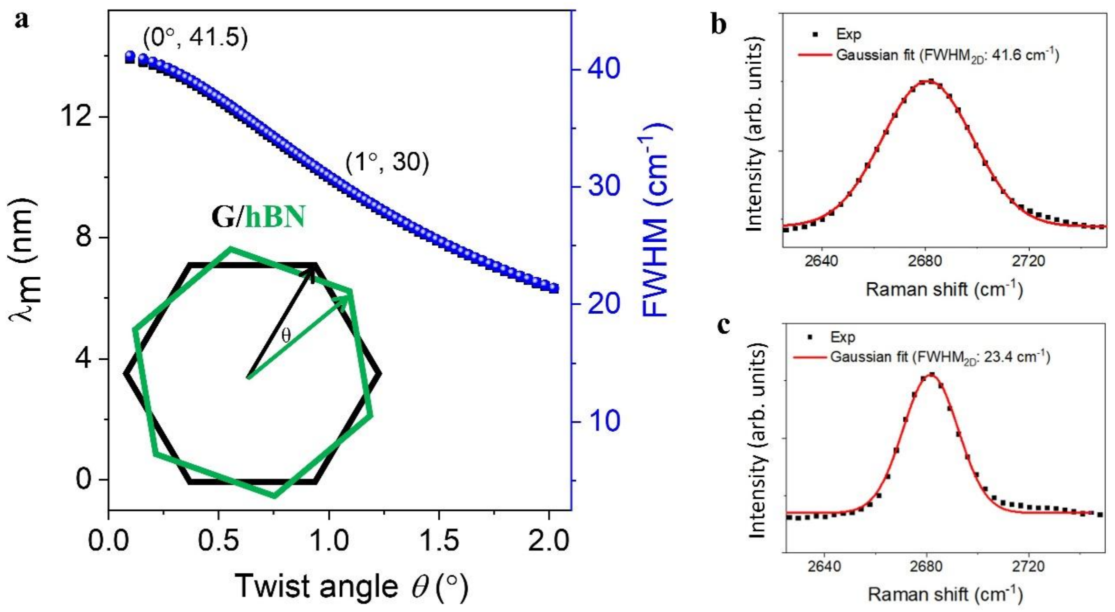

Supplementary Figure 2. Raman spectra of 0° G/hBN and 30° G/hBN. a, The relationship between FWHM and twist angle within 2°, coming from [1]. The FWHM of Raman 2D band is 41.5 cm^{-1} for a perfect alignment of 0°, where the FWHM is analyzed by Gaussian fitting. When the twist angle increases to 1°, the FWHM decreases to 30 cm^{-1}. This feature can be quickly used to identify graphene superlattices with a misalignment angle smaller than 2°. **b**, Raman spectra of G1/hBN, and the Gaussian fit of Raman 2D band is 41.5 cm^{-1}, indicating the twist angle between G1 and hBN is 0°. **c**, Raman spectra of G2/hBN, and the Gaussian fit of Raman 2D band is 23.4 cm^{-1}. Since the G1 and G2 are rotating 30°, thus the twist angle between G2 and hBN is 30°.

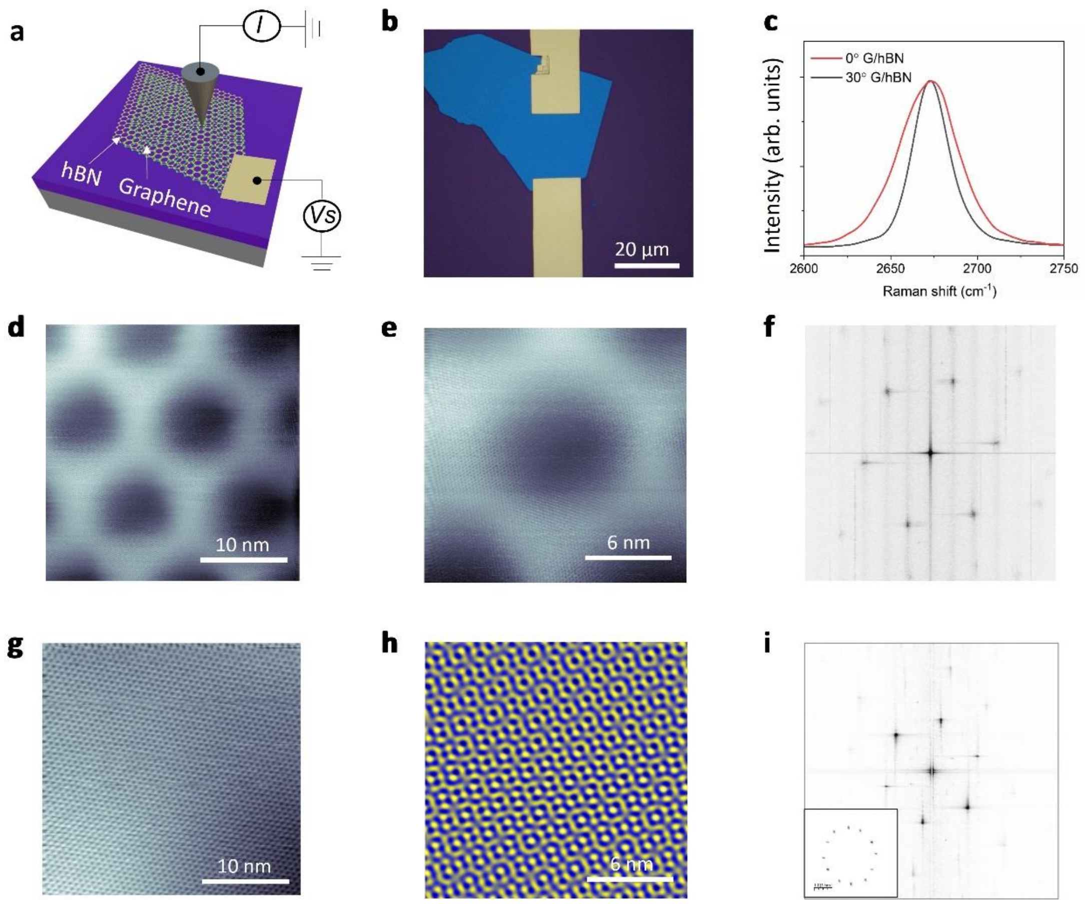

Supplementary Figure 3. Surface topography of 0° G/hBN and 30° G/hBN moiré superlattice. a, Schematic device set-up showing the moiré pattern of graphene on hBN. **b,** Optical image of G/hBN device for STM measurement. **c,** Raman 2D-band of 0° and 30° G/hBN superlattice for STM device. **d**, STM topographic images image (1500 mV, 15 pA) of 0° G/hBN device, a moiré pattern produced by graphene on hBN can be seen. **e**, The zoom-in STM image (500 mV, 50 pA) on an 18 nm region in **d** with a scale bar of 6 nm. **f**, Fourier transform of **d** showing the six Bragg peaks of the graphene lattice. **g**, STM topographic image (100 mV, 100 pA) of 30° G/hBN device, showing a smaller moiré pattern. **h,** Selected inverse Fourier transform of the moiré lattice. **i**, Fourier transform of **g** showing the atomic lattice as well as the moiré pattern. The scale bar is 5 nm^{-1}. Inset shows the highlights of the inner 12 points that correspond to the quasicrystal moiré lattice.

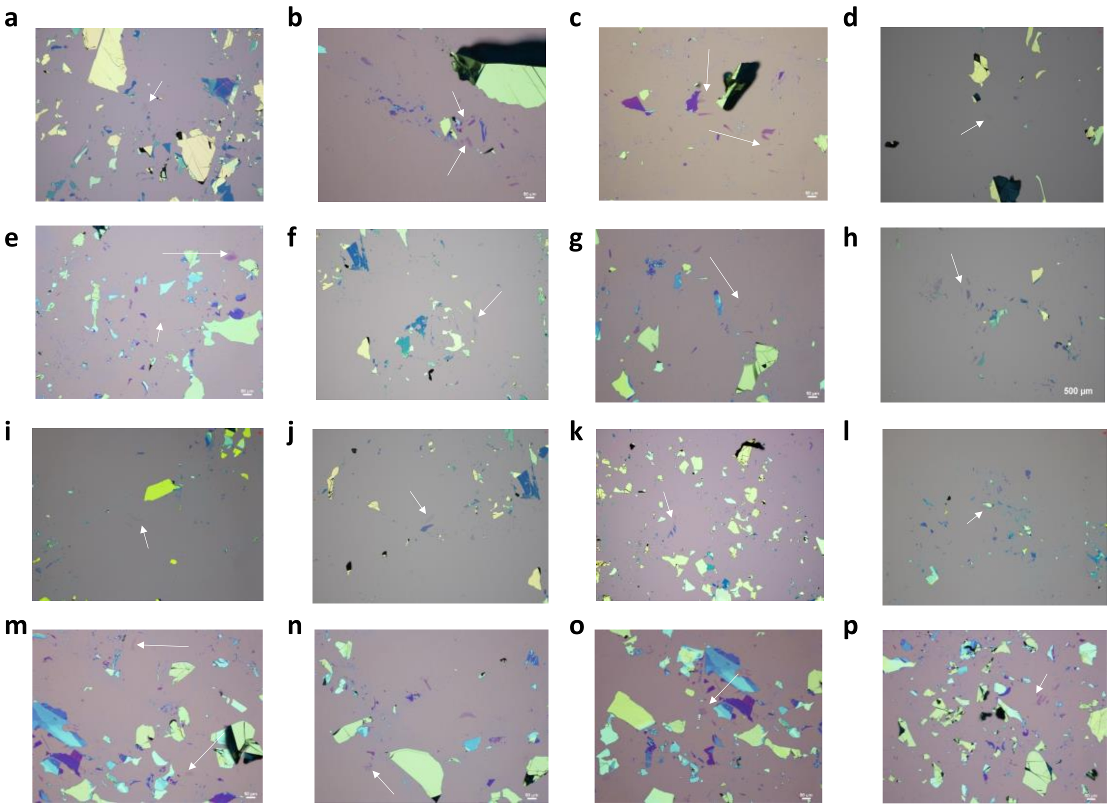

Supplementary Figure 4. Optical images of exfoliated graphene flakes on a silicon wafer. The single-layer graphene flakes are marked by white arrows. For the alignment of graphene and hBN, another challenge is to search for a single-layer graphene fake with a long and straight edge which can be used to identify the PCA. Usually, the proportion of single-layer graphene flakes only is 5-10% in all exfoliated flakes, even assisted by oxygen plasma and heating treatment. While in these sing-layer graphene flakes, most of the graphene flake has random shapes, and the graphene with a long straight edge can be used for alignment less than 10%. Consequently, these limitations make the desired graphene flakes that can be used for alignment less than 1%.

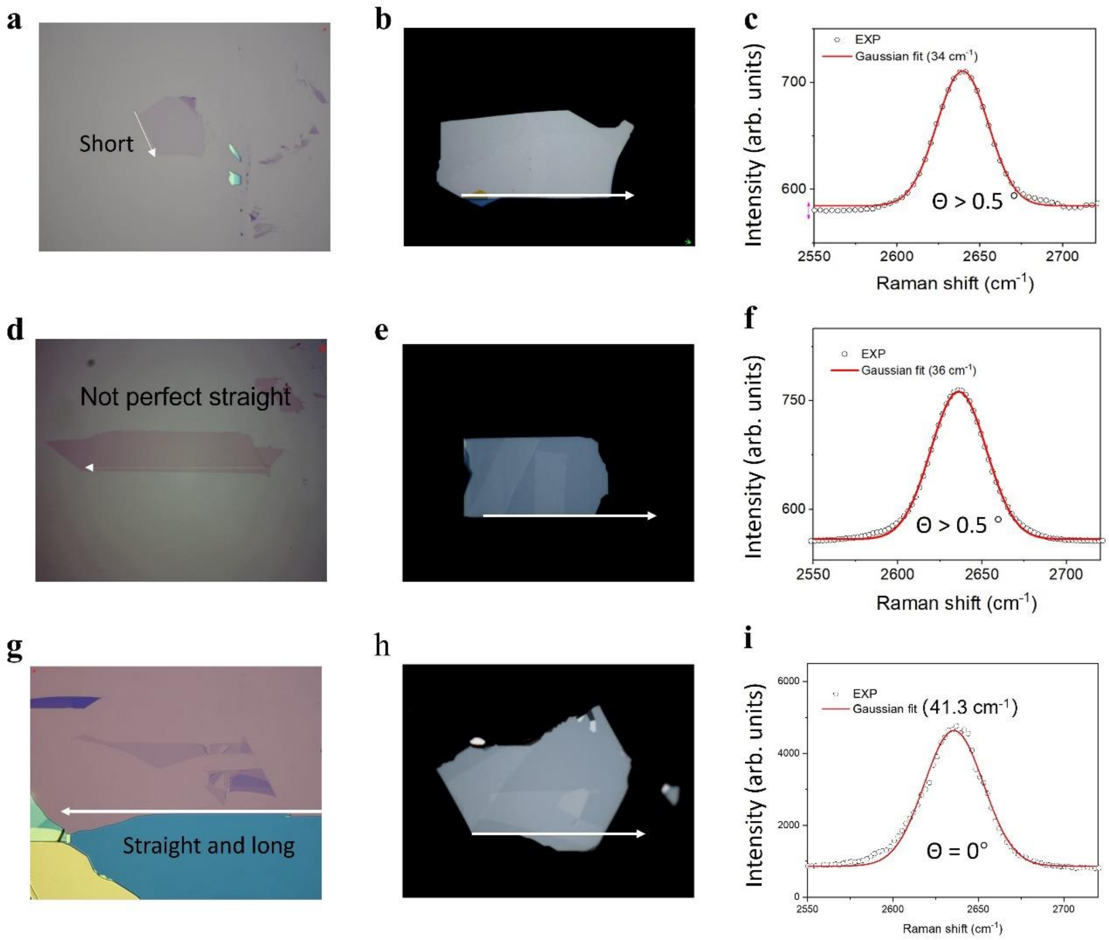

Supplementary Figure 5. Optical alignment using single-layer graphene and neighboring graphite edge. a, Optical image of graphene flake with short edge. **b**, Alignment of hBN and graphene using the graphene flake of **a**. **c**, Typical Raman 2D-band spectra. The Gaussian fit is close to 34.6 cm^{-1}, indicating the twist angle between graphene larger than 0.5°**. d,** Optical image of graphene flake with non-straight edge. **e**, Alignment of hBN and graphene using the graphene flake of **d**. **f**, Typical Raman 2D-band spectra. The Gaussian fit is 36.2 cm^{-1}. **g,** Optical image of graphite flake with straight and long edges. **h**, Alignment of hBN and graphene using the graphene edge of **g. i**, Typical Raman 2D-band spectra. The Gaussian fit is 41.3 cm^{-1}. According to the Raman determination of twist angle in Fig. S2, the FWHM of Raman 2D peak is 41.4 cm^{-1} for a perfect alignment of 0°. When the twist angle further increases to 0.5°, the FWHM decreases to 37.5 cm^{-1}. The FWHM of **c** and **f** is smaller than 37.5 cm^{-1}, which means the twist angles in **c** and **f** are larger than 0.5°. In contrast, the twist angle in **i** is close to 0°, indicating the perfect alignment using the neighboring graphite edge.

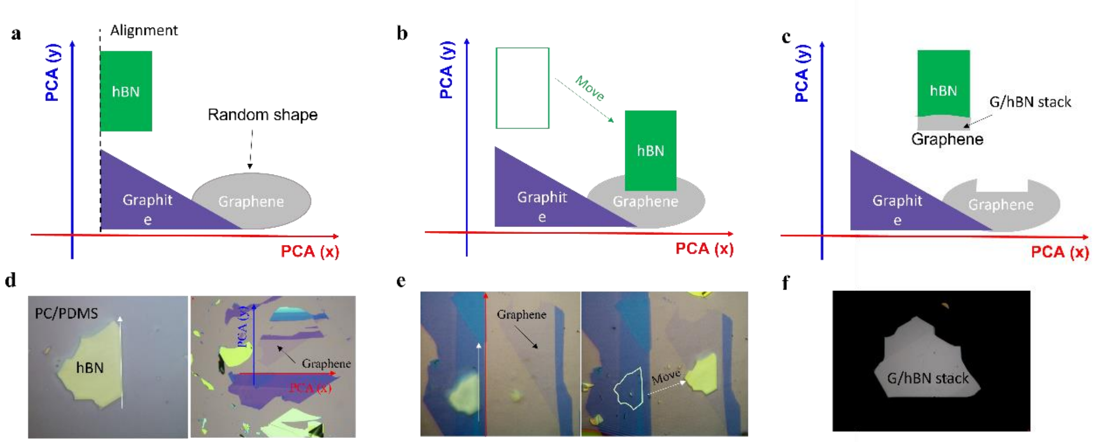

Supplementary Figure 6. Illustration of alignment using the neighboring graphite edge. a,d the hBN is aligned with PCA of the neighboring graphite edge, while the graphene itself can be any random geometric shape. Since graphene is connecting with neighboring graphite edge, so they can share the same PCA. **b,e** After alignment, the hBN moves to the position of graphene. **c,f**, the hBN is picking up graphene. The graphene will be detached to hBN due to the vdW interaction between graphene and hBN. **a,b,c** are the schematic cartoons, and **d, e, f,** are corresponding optical images. The best advantage of the current concept is that we do not need to consider the exact geometry of graphene itself, even though the circle shape of graphene as illustrated here, the round graphene can still be used to fabricate the moiré samples, significantly decreasing the difficulty of sample preparation.

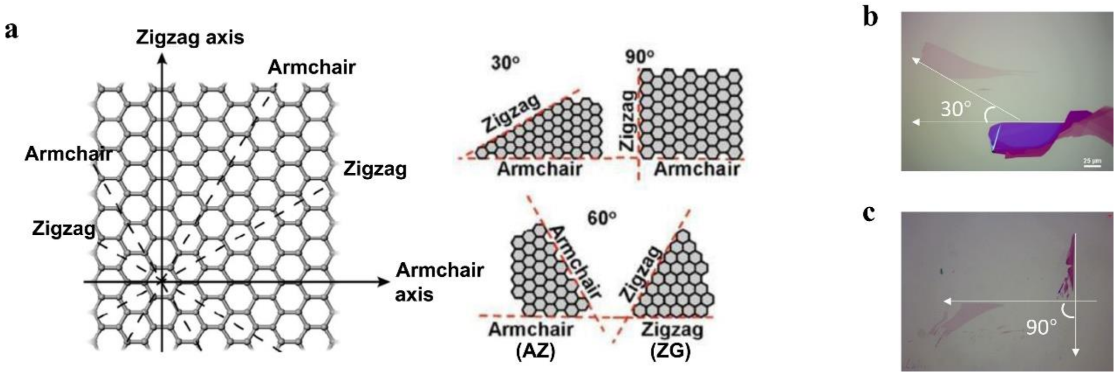

Supplementary Figure 7. The adjacent graphene flakes share the same PCA. a, Illustration of the relationship between angles and the chirality of the adjacent edges, adapted from [2]. Optical images of adjacent graphene flakes with an angle of **b**, 30 degrees and **c**, 90 degrees. Because both Graphene and hBN flakes have hexagonal symmetries, when the angle of the adjacent edge is an integer multiple of 30°, these two flakes usually come from the same crystal. This is because if they do not come from the same crystal, the probability of its edge oriented at an integer multiple of 30 degrees (n × 30°, n = 0, 1, 2 …12) is quite low, i.e., 12/360 (3.3%). In most probability, they are coming from the same crystal.

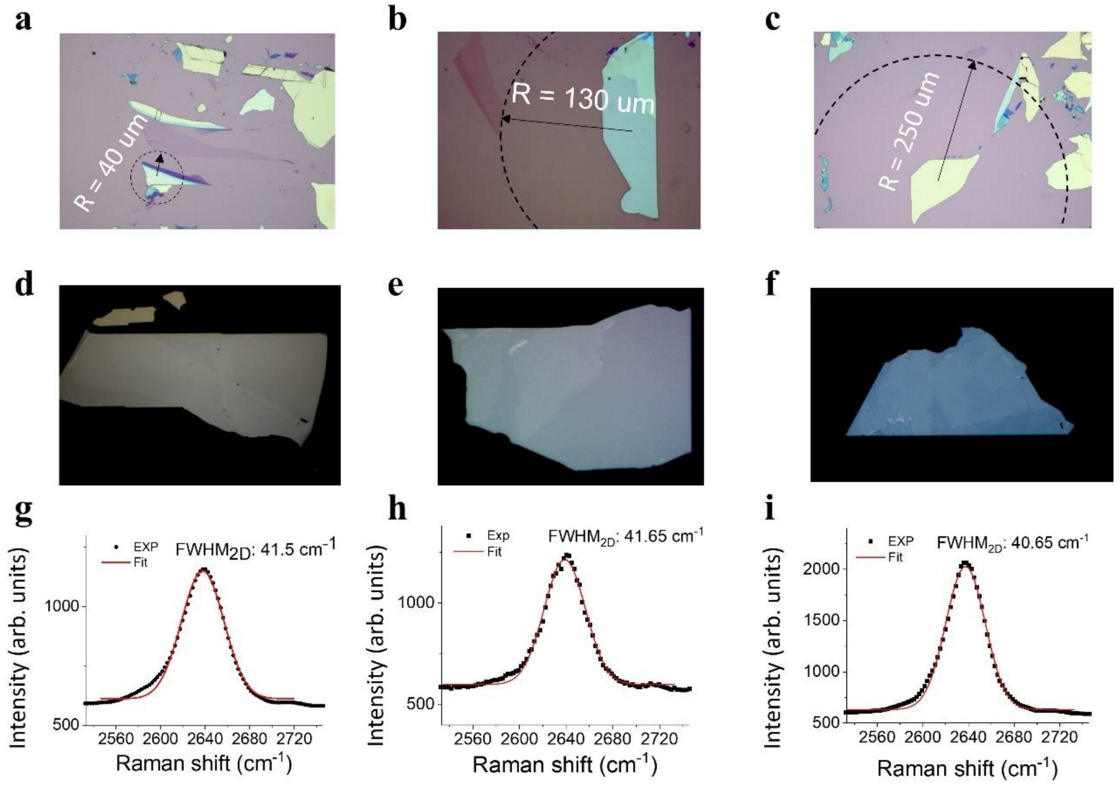

Supplementary Figure 8. The influence of the distance between graphene and graphite on the alignment. a-c, Optical images of graphene flakes with a distance of 40, 130 and 250 μm. **d-f**, The graphene/hBN stack using the 30° rotation technique. **g-i**, Typical Raman data from d-f. Regarding the distance between graphene and graphite, we have studied three different cases with a distance of 40, 130 as well as 250 μm. We found that in all three cases, the neighboring graphite edge can be used for alignment, as the Raman 2D peak shows that they are all close to 41 cm^{-1}. Therefore, we strongly recommend that potential users utilize our exfoliation method to ensure that the adjacent graphene and graphite share the same PCA.

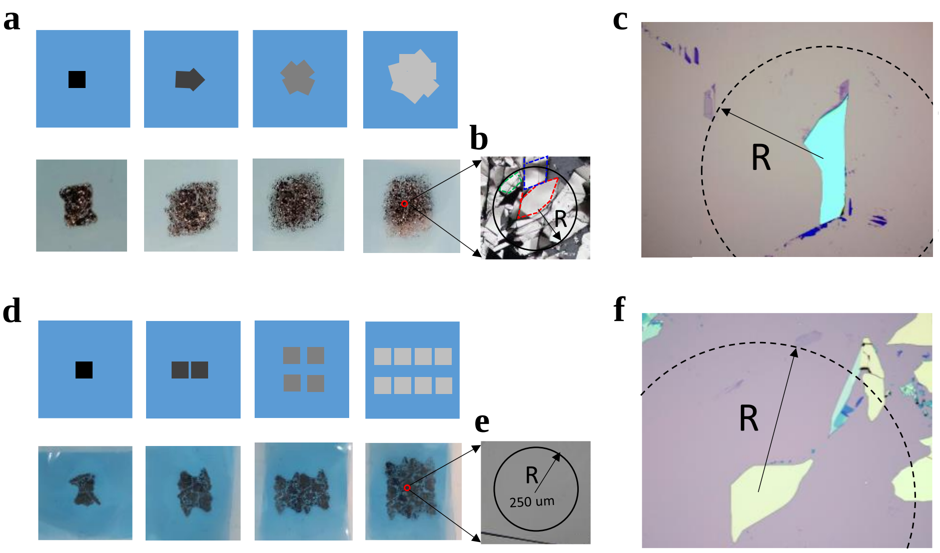

Supplementary Figure 9. Two methods of exfoliation graphene flakes. a-c, Overlap exfoliation. **d-f**, Non-overlap exfoliation. Generally, there are two kinds of exfoliation methods. One is the “overlap exfoliation” method. In this case, it certainly will not always be the case that adjacent graphene and graphite will share the same PCA because two adjacent crystals can be at any random orientation. However, we use another method called "non-overlap exfoliation”. In this method, we carefully exfoliate the graphene flakes multiple times and ensure the two graphite crystals are large and do not overlap with each other. In this case, in principle, within a radius R, their PCA should be the same.

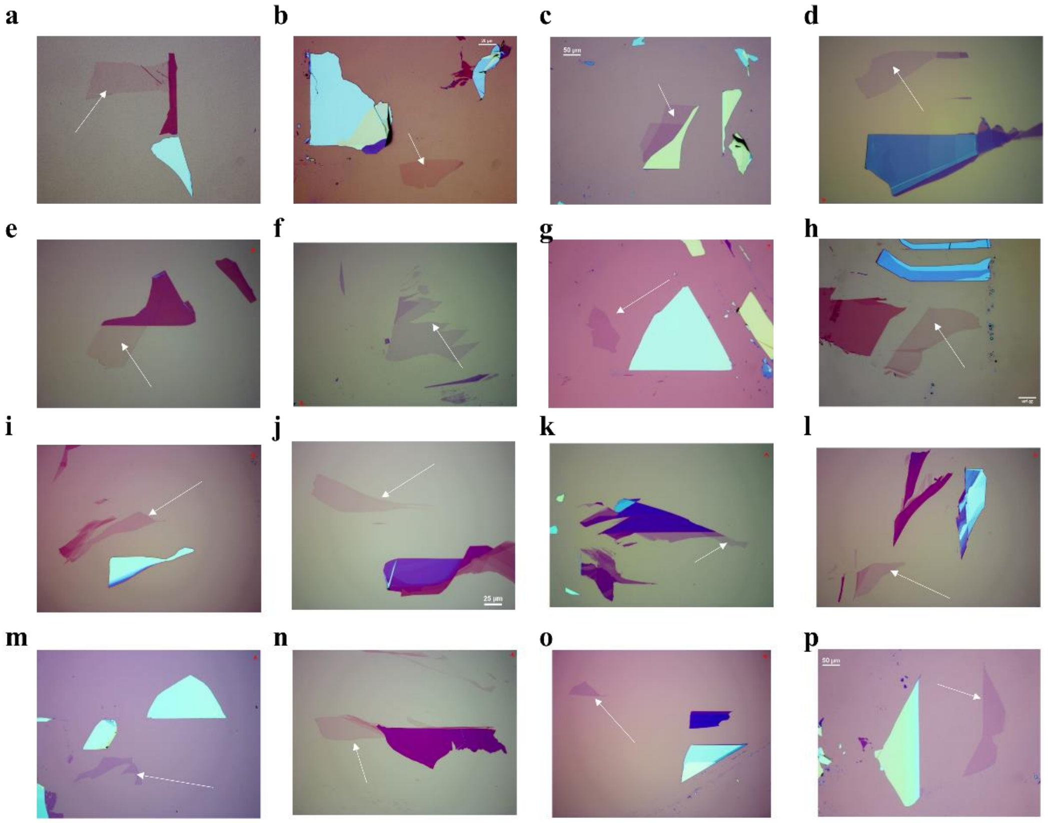

Supplementary Figure 10. Optical images of single-layer graphene without a straight edge. For conventional optical alignment, the graphene should have a straight edge for alignment. Therefore, the flakes in Fig. S10 cannot be considered for alignment. However, using our technique, the single-layer graphene itself does not have to have a straight edge since we can still use the neighboring graphite edge for fabricating moiré samples.

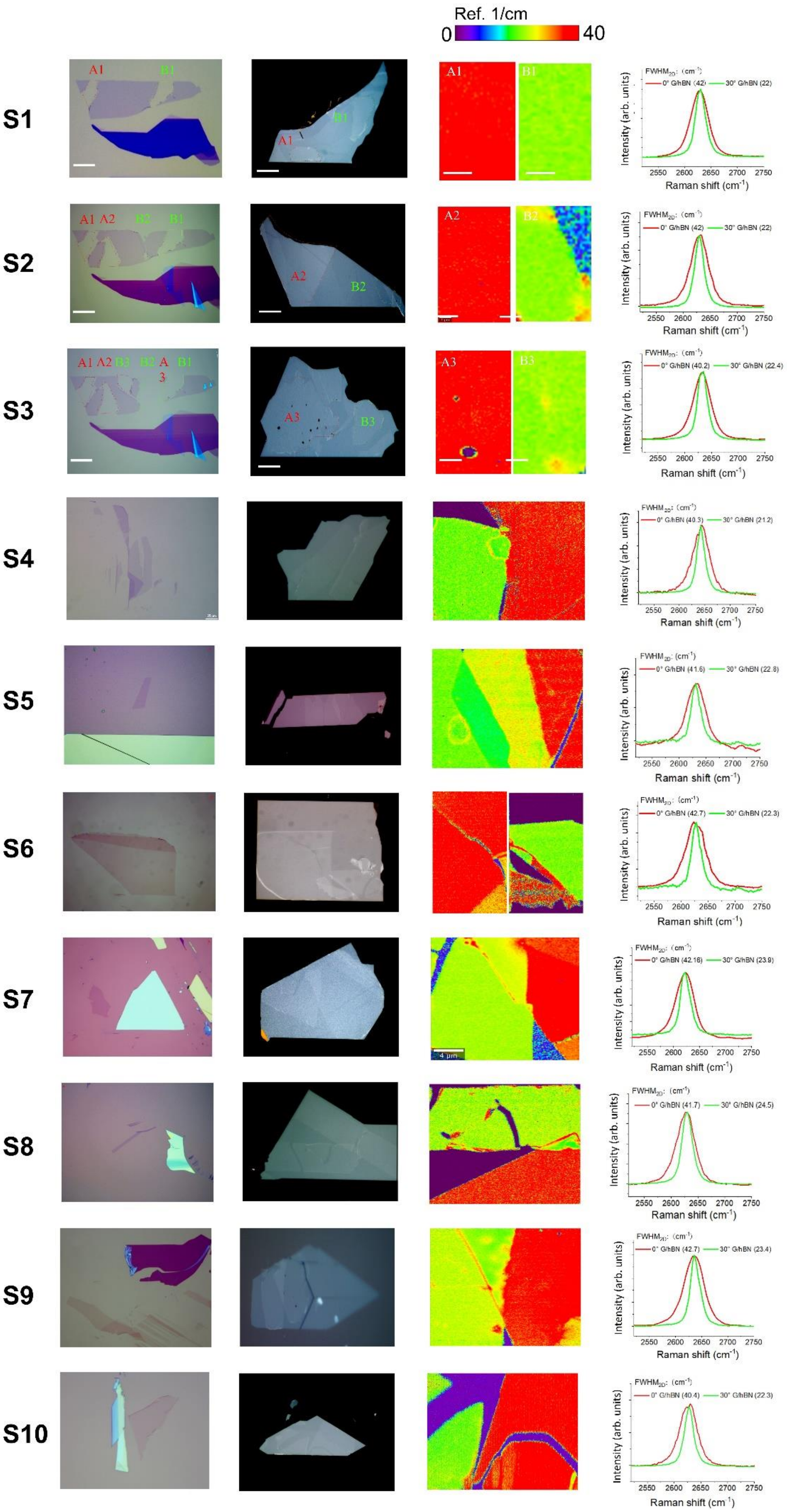

Ref. 1/cm
0
40
S1
S2
S3
S4
S5
S6
S7
S8
S9
S10
A1
B1
A1 A2 B2 B1
A2
B2
A1 A2 B3 B2 A 3 B1
A3
B3
4 μm
FWHM2D: (cm-1)
0° G/hBN (42) 30° G/hBN (22)
0° G/hBN (42) 30° G/hBN (22)
0° G/hBN (40.2) 30° G/hBN (22.4)
0° G/hBN (40.3) 30° G/hBN (21.2)
0° G/hBN (41.6) 30° G/hBN (22.8)
0° G/hBN (42.7) 30° G/hBN (22.3)
0° G/hBN (42.16) 30° G/hBN (23.9)
0° G/hBN (41.7) 30° G/hBN (24.5)
0° G/hBN (42.7) 30° G/hBN (23.4)
0° G/hBN (40.4) 30° G/hBN (22.3)
Intensity (arb. units)
Raman shift (cm-1)
2550 2600 2650 2700 2750

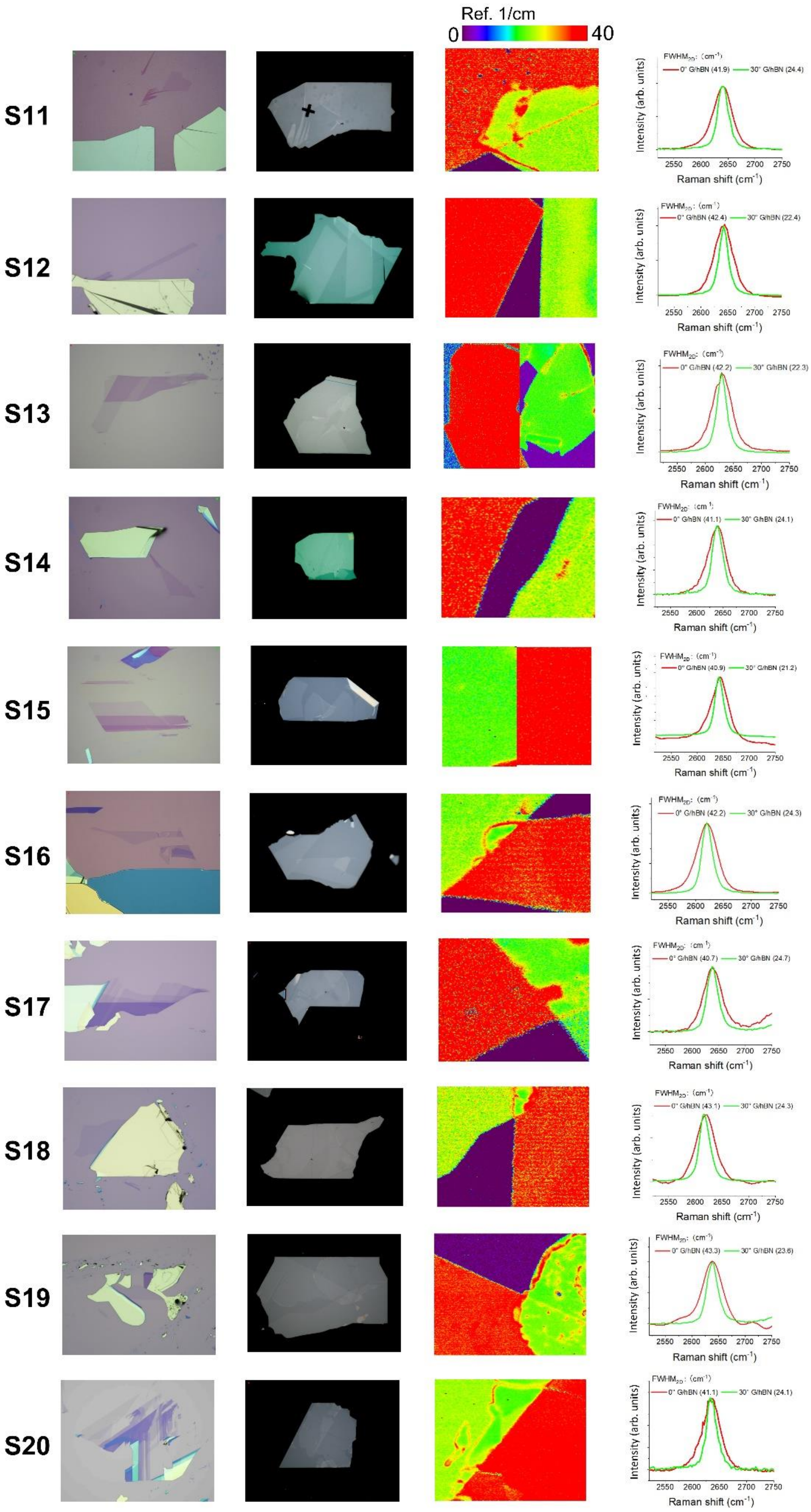
Ref. 1/cm
0
40
S11
FWHM2D: (cm-1)
0° G/hBN (41.9)
30° G/hBN (24.4)
S12
FWHM2D: (cm-1)
0° G/hBN (42.4)
30° G/hBN (22.4)
S13
FWHM2D: (cm-1)
0° G/hBN (42.2)
30° G/hBN (22.3)
S14
FWHM2D: (cm-1)
0° G/hBN (41.1)
30° G/hBN (24.1)
S15
FWHM2D: (cm-1)
0° G/hBN (40.9)
30° G/hBN (21.2)
S16
FWHM2D: (cm-1)
0° G/hBN (42.2)
30° G/hBN (24.3)
S17
FWHM2D: (cm-1)
0° G/hBN (40.7)
30° G/hBN (24.7)
S18
FWHM2D: (cm-1)
0° G/hBN (43.1)
30° G/hBN (24.3)
S19
FWHM2D: (cm-1)
0° G/hBN (43.3)
30° G/hBN (23.6)
S20
FWHM2D: (cm-1)
0° G/hBN (41.1)
30° G/hBN (24.1)
Intensity (arb. units)
2550 2600 2650 2700 2750
Raman shift (cm-1)

Supplementary Figure 11. Characterizations of 20 moiré samples with 100% alignment. The first row is the optical image of single-layer graphene, where the graphene itself has no straight edge for alignment, but the neighboring graphite edge can be used for alignment. The second row is the G/hBN stack after alignment using the graphene flake from the first row. The third row is the FWHM map of Raman 2D band from the second row. The red area shows the 0° G/hBN, and the green area shows the 30° G/hBN. The fourth row is the typical Raman spectra of 0° G/hBN and 30° G/hBN. The FWHM of Raman 2D band for all the samples is larger than 40 cm^{-1}, where the FWHM is analyzed by Gaussian fitting. According to the Raman determination of the twist angle in Fig.S2, our alignment is better than 0.2°.

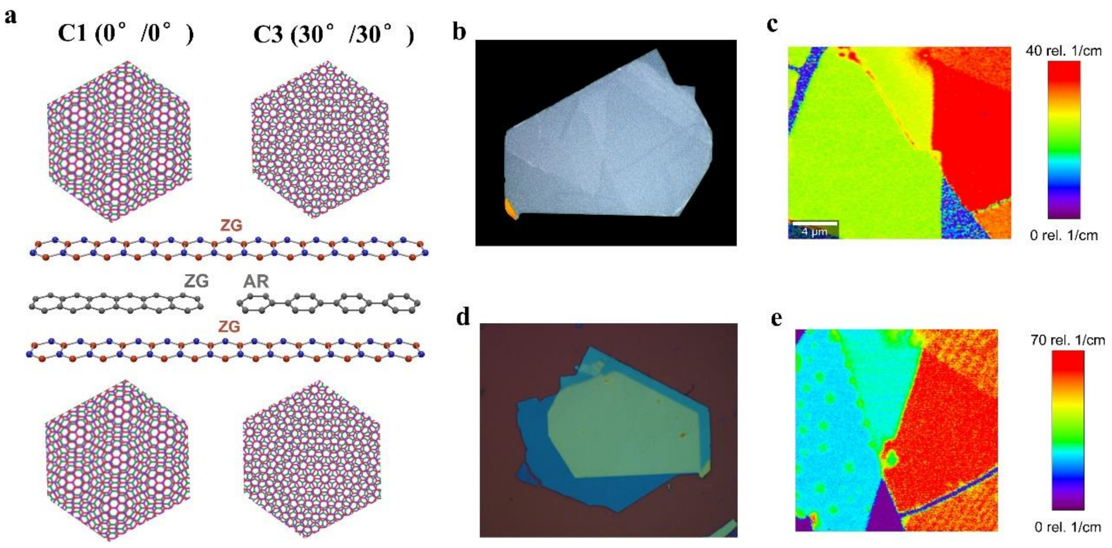

Supplementary Figure 12. Double alignment of T-hBN and B-hBN with same edge charity. a, Illustration of T-hBN and B-hBN with the same ZG edge, leading to the C1 (0°/0°) and C3 (30°/30°). **b**, Optical image of single alignment of graphene and hBN, with 0° and 30° G/hBN. **c**, Spatial map of the FWHM of 2D-band for the dashed area in **b**, indicating the 0° and 30° G/hBN. **d**, Optical image of T-hBN, graphene and B-hBN. **e**, Spatial map of the FWHM of 2D-band for the dashed area in **d**, purple area is 20 cm^{-1} and the red area is close to 70 cm^{-1}, indicating the C1 (0°/0°) and C3 (30°/30°). When T-hBN/G has the same edge charity as the B-hBN, the single 0° G/hBN will become double C1 (0°/0°), while the single 30° G/hBN will become double C3 (30°/30°).

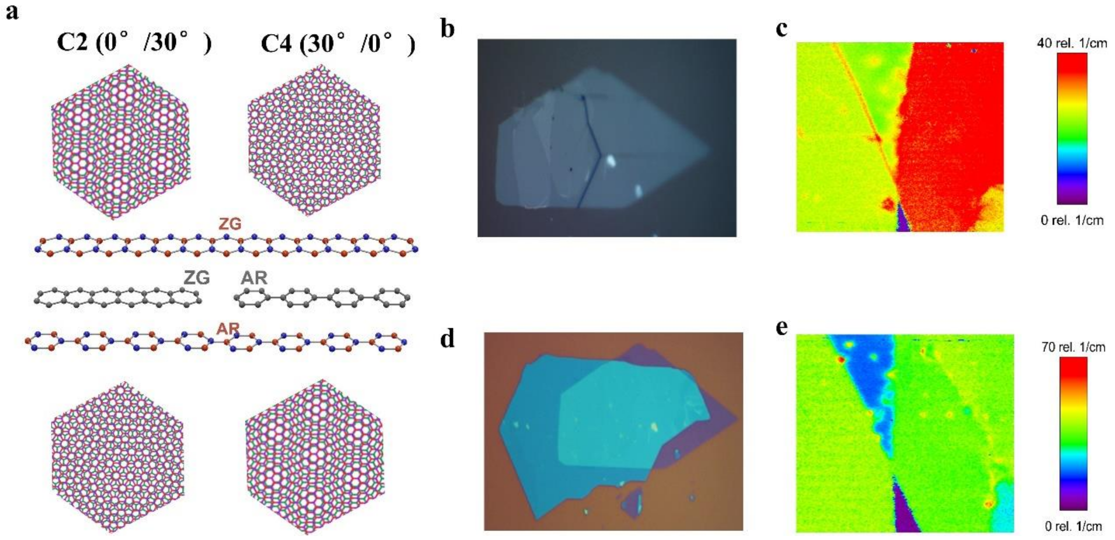

Supplementary Figure 13. Double alignment of T-hBN and B-hBN with different edge charity. a, Illustration of T-hBN and B-hBN with ZG edge and AR edge, respectively, leading to the C2 (0°/30°) and C4 (30°/0°). **b**, Optical image of single alignment of graphene and hBN, with 0° and 30° G/hBN. **c**, Spatial map of the FWHM of 2D-band for the dashed area in **b**, indicating the 0° and 30° G/hBN. **d**, Optical image of T-hBN, graphene and B-hBN. **e**, Spatial map of the FWHM of 2D-band for the dashed area in **d**, the green area is close to 40 cm^{-1}, indicating the of C2 (0°/30°) and C4 (30°/0°). When T-hBN/G has the different edge charity of the B-hBN, the single 0° G/hBN will still become single C2 (0°/0°), while the single 30° G/hBN will become single C4 (30°/0°).

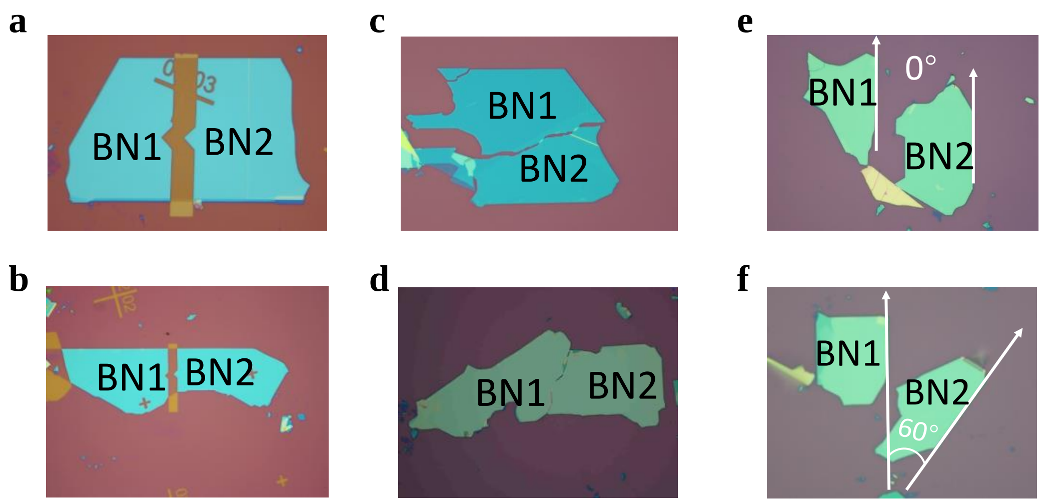

Supplementary Figure 14. Three possible cases of hBN used for flip-over technique. a,b, Optical images of two hBN crystals etched with DRIE into two isolated parts. **c,d,** Optical images of two fractured hBN after mechanical exfoliation. **e,f,** Optical images of adjacent hBN flakes with edge angle of 0 degree and 60 degrees. We can consider three different routes for obtaining BN1 and BN2 to be used during the flip-over technique. First, hBN can be cut into two (BN1 and BN2). In this case, we can make sure that BN1 and BN2 not only share the same PCA, but also the same surface. However, this cutting process is tedious, requiring complicated lithography steps. Second, BN1 and BN2 can come from naturally fractured hBN flakes, which can always be found during mechanical exfoliation**.** Third, we can also obtain BN1 and BN2 from two neighbouring hBN flakes whose PCA have integer multiples of 30 degrees to each other. The hBN also has a hexagonal symmetry. If the edge angle of the adjacent hBN is an integer multiple of 30°, then the two adjacent hBN flakes have a high chance of coming from the same crystal. This is because if they do not come from the same crystal, the probability of its edge oriented at an integer multiple of 30 degrees (n x 30°, n = 0, 1, 2 …11) is quite low, i.e., 12/360 (3.3%). In most probability, they come from the same crystal. Therefore, in this case, the two hBN flakes can also be used for alignment.

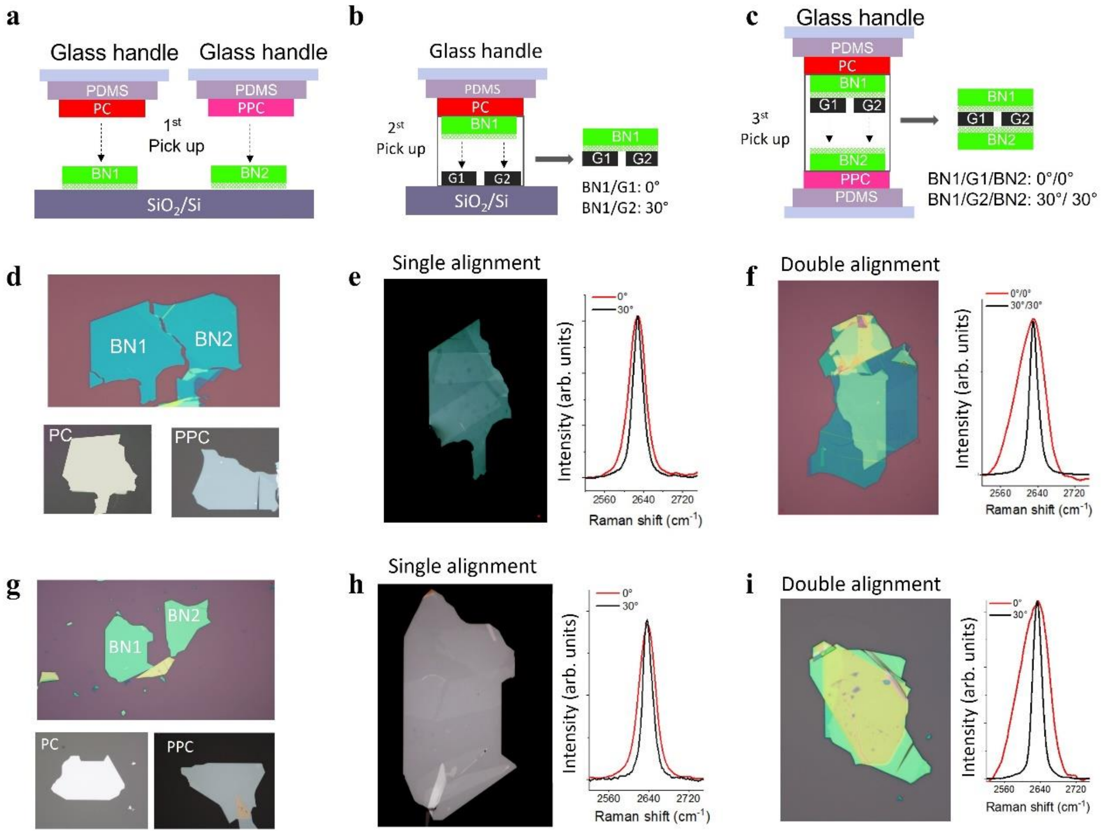

Supplementary Figure 15. Perfect double alignment using the flip-over technique. a,d,g PDMS/PC stamp and PDMS/PPC stamp pick up BN1 and BN2, respectively. Here we use PC and PPC because we can use PC pick up the BN2 from PPC, since the stickiness of PC is stronger than PPC. **b,e,h** Single alignment of BN1 with graphene, with rotating 30° each other. The Raman spectra in **e, h** confirm that there are 0° and 30° G/hBN stacks. **c**,**f**,**i**, BN1/G pick up the BN2 from PPC, and finally form an BN1/G/BN2 stack. The Raman spectra in **f, i** confirm that there are double 0° and double 30° G/hBN stacks. **a,b,c** are the schematic cartoons for double alignment, and **d, e, f,** are corresponding optical images and Raman spectrum for sample 1, and **g,h,i** are corresponding optical images and Raman spectrum for sample 2. Two hBN come from the same fractured hBN for sample 1 and for sample 2. The two hBN share the same PCA. Both these two cases can be used for controlling the lattice symmetry of T-hBN and B-hBN.

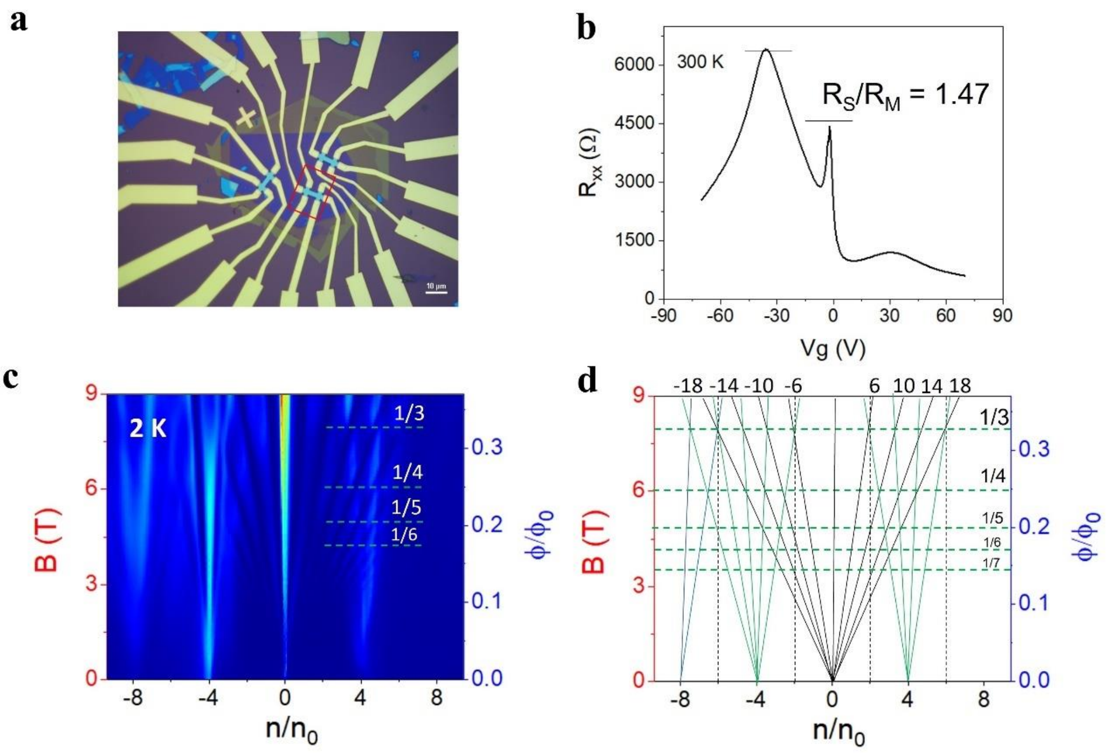

Supplementary Figure 16. Perfect double alignment for the second device. a, Optical image of the second double-aligned device. **b**, R_{xx} with gate voltage at room temperature. The hole side satellite resistance (Rs) is 1.47 times higher than the resistance of the central main Dirac point (R_M) at 300 K. **c**, Color map of sample resistance R_{xx} as a function of carrier density and magnetic field. ϕ/ϕ_0 and n/n_0 are the normalized magnetic flux and carrier density, respectively. **d**, Wannier diagram labeling the quantum Hall states identified in (**c**). The solid lines show quantum oscillations emerging from the primary Dirac point (PDP) and second Dirac point (SDP), with ν = ±2, ±6, ±10, ... for PDP (black). The horizontal dashed lines and numbers on the right show the most prominent BZ oscillations, with different values of $\frac{\phi}{\phi_0 q}$ $(q = 3, 4{,}5\ et\ al\ )$,

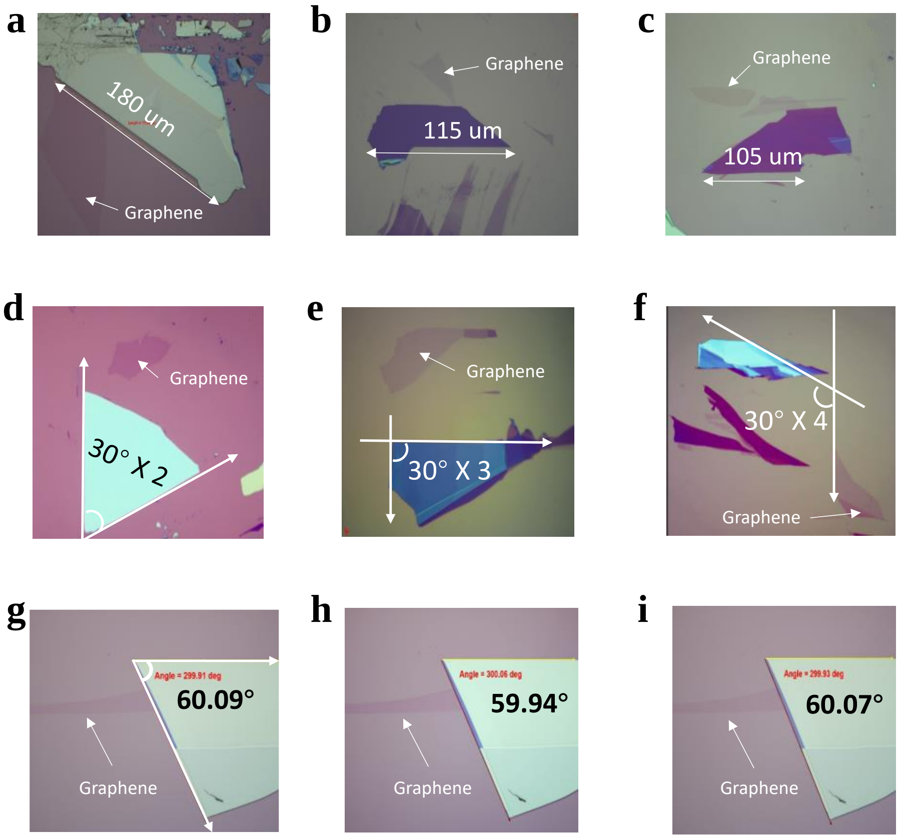

Supplementary Figure 17. Optical images of graphite for precisely representing the PCA. a-c, The length of the graphite edge is larger than 100 um. **d-f**, The angle of the graphite edge is integer multiples of 30 degrees. **g-i,** The error of three consecutive angle measurements Δθ is less than 0.2°.

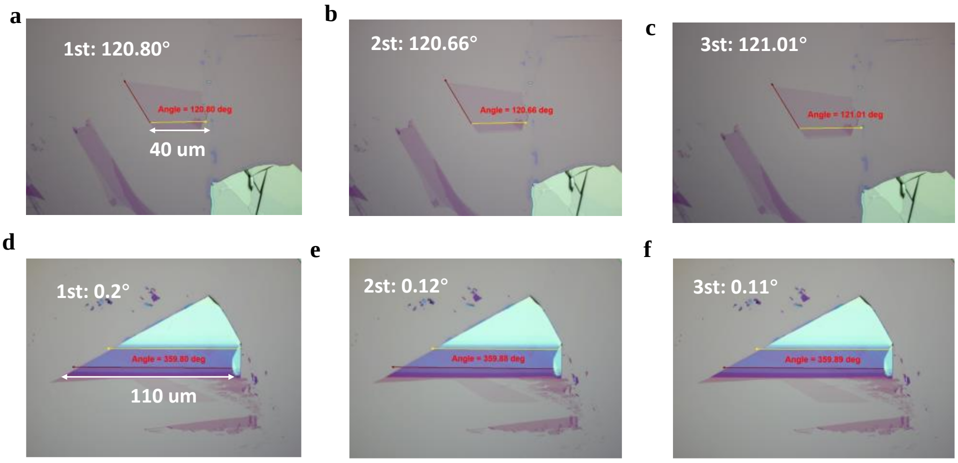

Supplementary Figure 18: Optical images of angle measurement with different edge lengths. The angle error of three consecutive measurements of **a-c**, $\Delta\theta > 0.5°$. **d-f**, $\Delta\theta < 0.2°$.

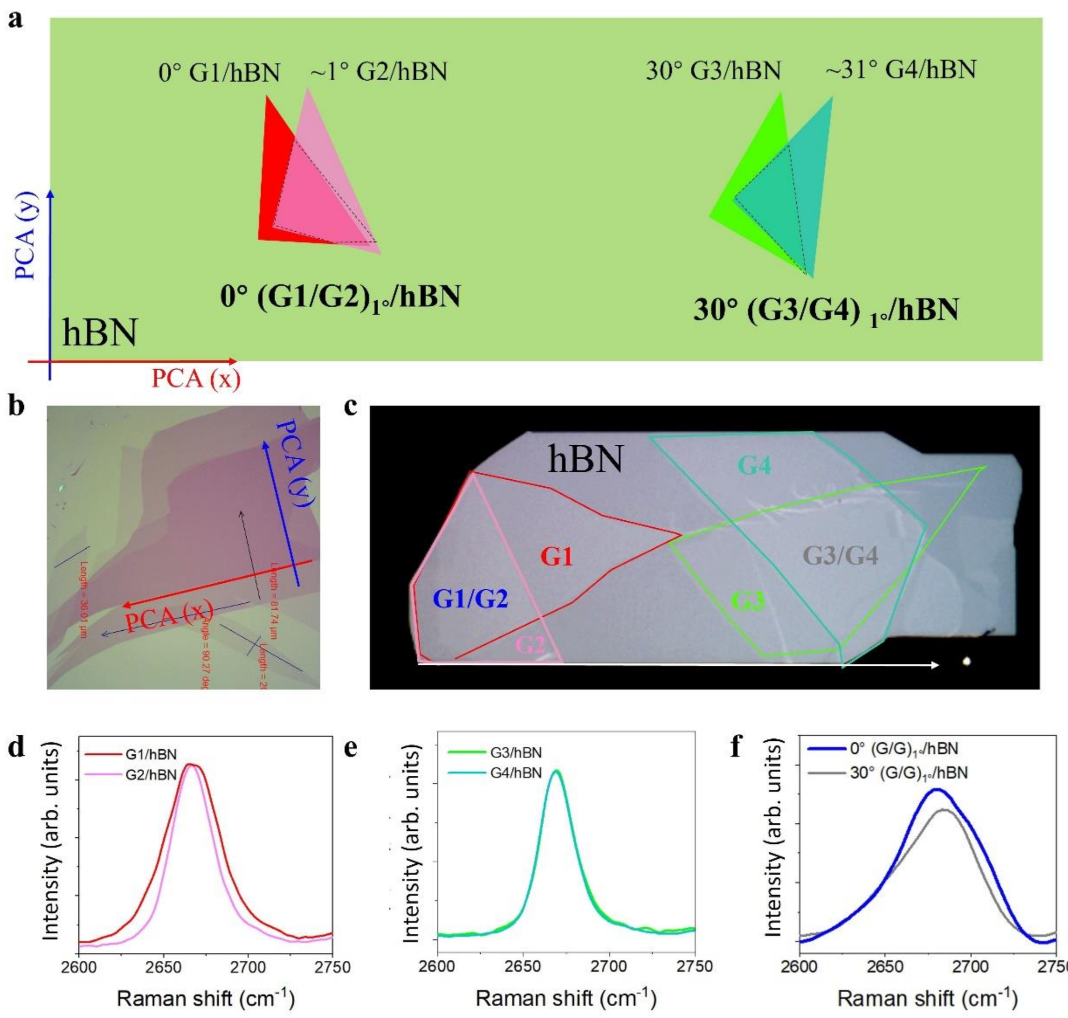

Supplementary Figure 19. Alignment of low-angle twisted bilayer graphene with hBN using our technique. a, Illustration of twisted bilayer graphene aligned with hBN. G1 edge aligned with the PCA of hBN, then G2 is rotating by ±1°. After this, G3 is rotating by 30° with PCA of hBN and G4 is rotating by ±1°. Using this concept, low angle twisted bilayer graphene of G1/G2 or G3/G4 must be 0° with hBN while the other must be 30° with hBN. **b**, Optical image of graphene with straight neighboring graphite edge used for PCA. **c**, The G/hBN stack shows four regions, using the concept in **a**. **d**, Raman spectra of G1 and G2, with Gaussian fitting of 41 and 32 cm^{-1}, indicating the G1 is nearly 0° aligned with hBN, while G2 is nearly 1° aligned with hBN. **e**, Raman spectra of G3 and G4, show similar FWHM feature of 23 cm^{-1}. **f**, Raman spectra of overlap area G1/G2 with FWHM of 58 cm^{-1} and G3/G4 with FWHM of 52 cm^{-1}. According to the relationship between wavelength and FWHM with the twist angle in Fig. S2, when the twist angle is close to 0°, the FWHM is very sensitive to the twist angle. While the twist angle is close to 30°, the FWHM changes slowly with the twist angle.

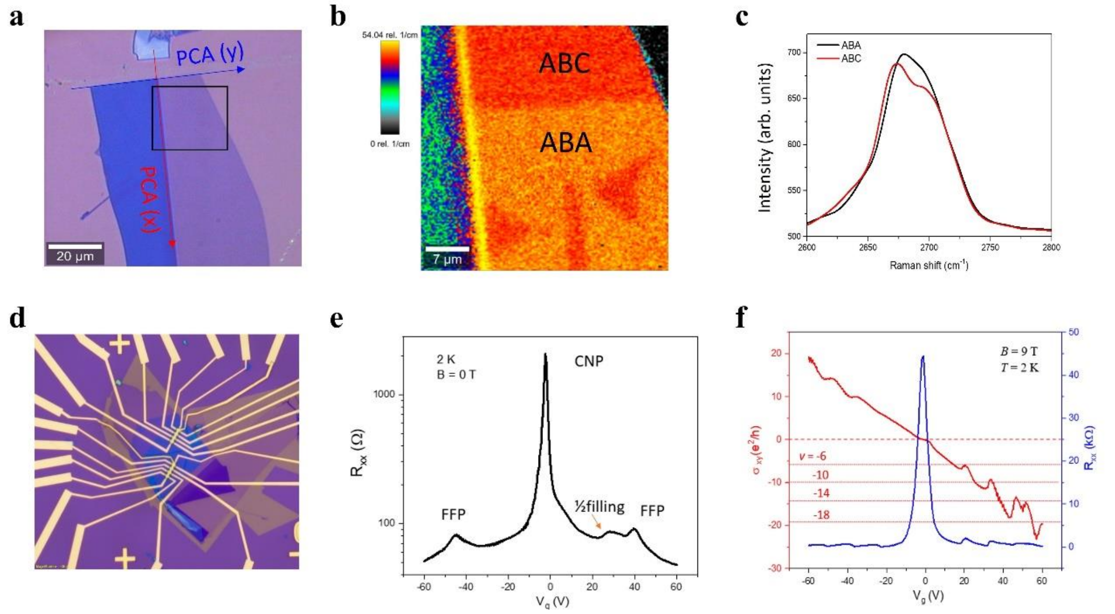

Supplementary Figure 20. Alignment of ABC-stacked trilayer graphene with hBN using our technique. a, Optical image of three-layer graphene with neighboring graphite edge for PCA. **b**, Spatial map of the FWHM of 2D-band for the dashed area in **a**, the deep red color region is ABC stacking domain, while the orange region is ABA staking domain. **c**, Typical Raman 2D-band of ABA and ABC trilayer graphene. **d**, Optical image of ABC G/hBN device. **e,** Longitudinal resistivity, R_{xx} (right scale), and Hall conductivity, $\sigma_{xy} = \rho_{xy}/(\rho_{xx}^2 + \rho_{xy}^2)$ (left scale) at B = 9 T and 2 K. Vertical arrows with numbers show LL filling v for the corresponding quantum Hall states. The well-resolved quantum Hall states at filling factors = 6, 10, 14, 18, etc. unambiguously confirm the ABC stacking order in trilayer graphene device [3]. **f**, Back gate dependent resistance R_{xx} at 0 T and 2K. In addition to a large peak at charge neutral peak (CNP) at 0 V, there are two extra prominent peaks symmetrically around CNP at a gate voltage of 44 V. Since they arise from fully filled moiré superlattice minibands, and we denote them as fully filled points (FFP). The emerging feature that distinguishes the ABC-TLG/hBN heterostructure is the extra prominent resistance maxima in the partially filled electron miniband, including a resistance peak close to 1/2 filling. Such a resistance peak close to 1/2 filling implies strongly correlated electronic states in our ABC-G/hBN moiré superlattice [4].

Structure	θ (Degree)	λ (nm)	n_s ($10^{12}\ cm^{-2}$)	Reference
hBN/Graphene/hBN (Single alignment)	0.3	13.4	n/a	[6]
	0.62	12	3	[7]
	0.43-1.14	9.2-12.9	4.5-2.78	[8]
	0.62	12	3	[9]
	0-1.5	8-14	n/a	[10]
	1	10	4.63	[11]
	0.5-1	10-12	n/a	[12]
	0.29-0.76	11.2-13.6	3.5-2.5	[13]
	0.4	12.9	2.5	[14]
	1	10	n/a	[15]
	0.6	12.06	3.3	[16]
	0-0.3	11.2-15.1	2-3.7	[17]
	0.85	10.7	3.5	[18]
	0 ± 0.02	14±0.3	2.35 ±0.1	Our work

Supplementary Table 1. Summary of the single-aligned G/hBN moiré samples. The relationship between θ, λ and n_s meet: $\lambda = \frac{1.018a_{CC}}{\sqrt{2.036[1-cos(\theta)]+0.018^2}}$, $n_s = 4/A = \frac{8}{\sqrt{3}\lambda^2}$, where A is the size of moiré superlattice and a_{cc} is the lattice parameter of graphene crystal. the twist angle (θ) from the moiré full filling n_s has some error, because superlattice gaps are usually present over a range of n_s. Typically, we can identify the position of full filling to an accuracy of $\delta n_s \approx 10^{11}\ cm^{-2}$, corresponding to a twist angle error of ±0.02° [5].

θ_{Top} (°)	λ_{Top} (nm)	$n_{s,Top}$ (10^{12} cm^{-2})	θ_{Bottom} (°)	λ_{Bottom} (nm)	$n_{s,Bottom}$ (10^{12} cm^{-2})	Methods	Modes	Reference
0	n/a	n/a	0	n/a	n/a	AFM tip	Dynamic	[19]
0.24	14.7	2.15	0.38	14	2.34	PDMS hemisphere		[20]
0	15.3	1.98	0.4	14	2.34	Optical alignment	Stationary	[21]
0.34	13.2	2.4	1.2	9	5.2			[22]
0	14	2.35	0.45	12	2.3			[23]
0	13.7	2.46	0.56	12.2	3.09			[24]
0.73	11.3	3.6	1.10	9.9	4.7			[25]
0 ±0.02	14 ±0.3	2.35 ±0.1	0 ±0.02	14 ±0.3	2.35 ±0.1			Our work

Supplementary Table 2. Summary of the double-aligned hBN/G/hBN supermoiré samples. Note: θ_{Top} and θ_{Bottom} are the twist angles between graphene and the top/bottom hBN; λ_{Top} and λ_{Bottom} are the moiré wavelength between graphene and the top/bottom; $n_{s,Top}$ and $n_{s,Bottom}$ are the carrier density to fill the mini Brillouin zone in the top/bottom moiré superlattice.

Technical indicators	Traditional technique	Our technique
Alignment yield	8% (12.5%)	>90% (100%)
Precision	0.5-1 degree	<0.2 degree
Fabrication time	10-20 hours/1 sample	1-2 hours/ 1 sample

Supplementary Table 3. Summary of the alignment yield, precision, and fabrication time using our technique. We would like to summarize the improvement in alignment yield, fabrication time and precision offered by our technique. Alignment yield. The success rate demonstrated by an earlier work [23] using the traditional random stacking method was only ~8% (5 successful samples out of 63 devices), which is close to the theoretical limitation of 1/8 (12.5%). As long as our "Golden Rule of Three" is strictly followed, the success rate can be more than 90%, which is close to the theoretical value of 100%. The gap between experimental value and theoretical value can be made by the human errors since the transfer is highly dependent on personal experience and operation. Precision. Using a traditional method (Table S1), the achieved precision is typically around 0.5-1 degree. One typical example can be seen in our previous work [18], where the twist angle between graphene and hBN is as large as 0.85 degrees. Using our alignment technique and strictly following the "Golden Rule of Three" guarantee a precision of better than 0.2 degrees. Fabrication time. Traditionally, finding a single layer of graphene with a straight edge for the alignment process is time-consuming. Our technique proposes that neighbouring graphite can also be used for alignment. Timewise, we can confidently say our technique is 10 times more efficient on average compared to other traditional methods. From the table above, we can see that there is a clear improvement in device yield, precision and fabrication time using our technique.

Supplementary Note 1. Theoretical calculation of the total energy in relaxed graphene-hBN heterostructure.

Let $a\ (= 0.142\ nm)$ be the C-C bond length of graphene, the lattice vectors are given by

$$\vec{a}_1 = \left(\sqrt{3}a, 0\right) \qquad \vec{a}_2 = \left(\frac{\sqrt{3}}{2}a, \frac{3}{2}a\right)$$

Lattice constant mismatch between graphene and hBN is $\epsilon = \frac{a_G - a_{BN}}{a_{BN}} = -0.017$ and for relative rotation angle θ, the moiré lattice vectors are given by

$$\vec{A}_i = [(1+\epsilon)R(\theta) - \mathrm{I}]\vec{a}_i; \quad i = 1,2$$

With the moiré period

$$L_M = \frac{\sqrt{3}a}{\sqrt{\epsilon^2 + 2(1+\epsilon)\left(1 - \cos(\theta)\right)}}$$

Graphene reciprocal lattice vectors are given by

$$\vec{g}_m = \hat{R}_{\frac{2\pi(m-1)}{6}}\left(0, \frac{4\pi}{3a}\right),$$

Moiré reciprocal lattice vectores are related by

$$\vec{G}_m = [(1+\epsilon)R^{-1} - \mathrm{I}]\vec{g}_m$$

Due to coupling between two layers, the atoms tend to move around in order to minimize the total energy. The local displacement of the atoms in the continuum limit is given by

$$\vec{u}(\vec{r}) = \frac{k_s|\epsilon|^3}{G^2\tilde{\epsilon}^2}[\vec{\nabla}_r + \frac{\chi_R}{\epsilon}\sin(\theta)\,\hat{z}\times\vec{\nabla}_r]\left(U_1 f_1(\vec{r}) + U_2 f_2(\vec{r})\right)$$

Where

$$\tilde{\epsilon} = \sqrt{\epsilon^2 + 2(1+\epsilon)(1 - \cos(\theta))}$$

$$k_s = \frac{2}{3\sqrt{3}\,\epsilon^2 a^2(\lambda_g + 2\mu_g)} = 0.029\ meV^{-1}$$

$$\chi_R = 2 + \frac{\lambda_g}{\mu_g} = 2.34$$

$$\left(f_1(\vec{r}), f_2(\vec{r})\right) = \sum_m (1,\ \ i(-1)^{m-1}) \exp\left(i\, \vec{G}_M \cdot \vec{r}\right)$$

The elastic and potential energies are given by the integrals

$$E_e = \int_{A_M} d\vec{r}\, U_e(\vec{r})\,, E_p = \int_{A_M} d\vec{r}\, U_p(\vec{r})$$

where the energy densities are given by

$$U_e(\vec{r}) = \frac{1}{2}(\lambda_g + \mu_g)\left(u_{xx} + u_{yy}\right)^2 + \frac{\mu_g}{2}\left[\left(u_{xx} - u_{yy}\right)^2 + \left(2u_{xy}\right)^2\right]$$

$$U_p(\vec{r}) = \frac{1}{A_g}\left[U_0 + U_1\tilde{f}_1(\vec{d}) + U_2\tilde{f}_2(\vec{d})\right]$$

Where $u_{ij} = \frac{1}{2}(\partial_i u_j + \partial_j u_i)$ and $\left(\tilde{f}_1(\vec{d}), \tilde{f}_2(\vec{d})\right) = \sum_m (1, i(-1)^{m-1}) \exp\left[\, i\, \vec{g}_n \cdot \vec{d}\right)$; $\vec{d} = [(1+\epsilon)R_\theta - I]\vec{r} + \vec{u}(\vec{r})$, R_θ being the rotation matrix. Due to the hexagonal symmetry of the moiré lattice, energies for all angles can be found from energies for angles in the range $0^\circ \leq \theta < 30^\circ$. Calculated E_e, E_p and $E_{total} = E_e + E_p$ are shown as a function of relative rotation angles near 0° and 30° in Fig.1b and Fig.1c.

Supplementary Note 2. Continuum model of graphene moiré superlattice.

We used the continuum model approach to study the effect of the moiré potentials caused by both top and bottom hBN stacks on the electronic properties of monolayer graphene [26, 27]. The Hamiltonian for the moiré heterostructure is symbolically represented by the following 3×3 matrix, where each entry is by itself a 2x2 matrix:

$$H = \begin{pmatrix} H_G & T^\dagger & B^\dagger \\ T & H_{thBN} & 0 \\ B & 0 & HbhBN \end{pmatrix}$$

Where $H_G = -\hbar v k.\sigma_\xi$ is the Dirac Hamiltonian that describes graphene electronic states and $\xi = \pm 1$ is the valley index of graphene. $H_{thBN} = H_{bhBN} = diag(V_N, V_B)$ is the Hamiltonian that describes the effect of the top and bottom hBN stacks. T and B represent the moiré coupling matrices that couple electronic states of monolayer graphene with electronic states of the hBN stacks. In general, T and B are written as:

$$T = t\left[\begin{pmatrix} 1 & 1 \\ 1 & 1 \end{pmatrix} + \begin{pmatrix} 1 & \omega^{-\xi} \\ \omega^{\xi} & 1 \end{pmatrix} e^{i\xi G_1^T \cdot r} + \begin{pmatrix} 1 & \omega^{\xi} \\ \omega^{-\xi} & 1 \end{pmatrix} e^{i\xi (G_1^T + G_2^T)\cdot r}\right]$$

where t is the strength of the moiré potential energy $t = 150\, meV$ and $\omega = e^{i\frac{2\pi}{3}}$.
A similar expression is also written for B with the reciprocal lattice vector of the top stack G_i^T replaced by reciprocal lattice vectors of the bottom stack G_i^B. Both G_i^T and G_i^B are determined by the relative alignment between the monolayer graphene and the corresponding hbN stack.
The lattice vectors for monolayer graphene are:

$$a_1 = a(1,0)$$
$$a_2 = a\left(\frac{1}{2}, \frac{\sqrt{3}}{2}\right)$$

where a is the primitive lattice vector length of graphene, $a = 0.246\, nm$
The rotated lattice vectors of a given hBN stack are:

$$a_i^{T/B} = M\, R(\theta^{T/B}) a_i$$

where $R(\theta^{T/B})$ is the two-dimensional rotation matrix parametrized by the amount of twist angle for top θ^T and bottom θ^B hBN stacks. M is the stretch factor between

graphene and hBN lattices, given by $M = 1 + \epsilon = \frac{a_{hbNG}}{a_G} = 1.018$.

Finally, the primitive lattice vectors of single- moiré structures are:

$$L_i^{T/B} = \left[1 - R\left(\theta^{T/B}\right)^{-1} M^{-1}\right] a_i$$

and the corresponding k-space vectors are:

$$G_i^{T/B} = \left[1 - M^{-1} R\left(\theta^{T/B}\right)\right] b_i$$

where b_i are the primitive k-space vectors for monolayer graphene.

Supplementary References

[1] Eckmann, A. et al. Raman fingerprint of aligned graphene/hBN superlattices. Nano Lett. **13,** 5242-5246 (2013).
[2] You, Y. M. et al. Edge chirality determination of graphene by Raman spectroscopy. Appl. Phys. Lett. **93,** 163112 (2008).
[3] Zhang, L., Zhang, Y., Camacho, J. et al. The experimental observation of quantum Hall effect of l=3 chiral quasiparticles in trilayer graphene. Nature Phys **7,** 953–957 (2011).
[4] Chen, G., Jiang, L., Wu, S. et al. Evidence of a gate-tunable Mott insulator in a trilayer graphene moiré superlattice. Nat. Phys. **15,** 237–241 (2019).
[5] Liu, X., Hao, Z., Khalaf, E. et al. Tunable spin-polarized correlated states in twisted double bilayer graphene. Nature **583,** 221–225 (2020).
[6] Yankowitz, M., Xue, J., Cormode, D. et al. Emergence of superlattice Dirac points in graphene on hexagonal boron nitride. Nature Phys **8,** 382–386 (2012).
[7] Ponomarenko, L., Gorbachev, R., Yu, G. et al. Cloning of Dirac fermions in graphene superlattices. Nature **497,** 594–597 (2013).
[8] Hunt, B. et al. Massive Dirac fermions and Hofstadter butterfly in a van der Waals Heterostructure. Science **340,** 1427 (2013).
[9] Gorbachev, R. V. et al. Detecting topological currents in graphene superlattices. Science **346,** 448 (2014).
[10] Woods, C., Britnell, L., Eckmann, A. et al. Commensurate–incommensurate transition in graphene on hexagonal boron nitride. Nature Phys **10,** 451–456 (2014).
[11] Lei Wang et al. Evidence for a fractional fractal quantum Hall effect in graphene superlattices., Science **350,** 1231 (2015).
[12] Woods, C., Withers, F., Zhu, M. et al. Macroscopic self-reorientation of interacting two-dimensional crystals. Nat Commun **7,** 10800 (2016).
[13] Kumar, R. K. et al. High-temperature quantum oscillations caused by recurring Bloch states in graphene superlattices. Science **357,** 181–184 (2017).
[14] Chen, G. R. et al. Emergence of tertiary Dirac points in graphene moiré superlattices. Nano Lett. **17,** 3576 (2017).
[15] Spanton, E. M. et al . Observation of fractional Chern insulators in a van der Waals

heterostructure. Science **360,** 62-66 (2018).
[16] Yankowitz, M., Jung, J., Laksono, E. et al. Dynamic band-structure tuning of graphene moiré superlattices with pressure. Nature **557,** 404–408 (2018).
[17] Wallbank, J.R., Krishna Kumar, R., Holwill, M. et al. Excess resistivity in graphene superlattices caused by umklapp electron–electron scattering. Nature Phys **15,** 32–36 (2019).
[18] He, P., Koon, G.K.W., Isobe, H. et al. Graphene moiré superlattices with giant quantum nonlinearity of chiral Bloch electrons. Nat. Nanotechnol. **17,** 378–383 (2022).
[19] Finney, N.R., Yankowitz, M., Muraleetharan, L. et al. Tunable crystal symmetry in graphene–boron nitride heterostructures with coexisting moiré superlattices. Nat. Nanotechnol. **14,** 1029–1034 (2019).
[20] Yang, Y. P. et al. In situ manipulation of van der Waals heterostructures for twistronics. Sci. Adv. **6,** eabd3655 (2020).
[21] Wang, Z. H. et al. Composite super-moiré lattices in double-aligned graphene heterostructures. Sci. Adv. **5,** eaay8897 (2019).
[22] Wang, L. J. et al. New Generation of moiré superlattices in doubly aligned hBN/Graphene/hBN heterostructures. Nano Lett. **19,** 2371−2376 (2019).
[23] Sun, X., Zhang, S., Liu, Z. et al. Correlated states in doubly-aligned hBN/graphene/hBN heterostructures. Nat Commun **12,** 7196 (2021).
[24] Kuiri, M. et al. Enhanced electron-phonon coupling in doubly aligned hexagonal boron nitride bilayer graphene heterostructure. Phys. Rev. B. **103,** 115419 (2021).
[25] Anđelković, M. et al. Double moiré with a twist: Supermoiré in encapsulated graphene. Nano Lett. **20,** 979–988 (2020).
[26] Rafi Bistritzer and Allan H. MacDonald, Moiré bands in twisted double-layer graphene, PNAS. **108,** 12233 (2011).
[27] Moon, P., Koshino, M. Electronic properties of graphene/hexagonal-boron-nitride moiré superlattice. Phys. Rev. B. **90,** 155406 (2014).
